# Self-evaluation and depression in adolescents with a chronic illness: A systematic review

**DOI:** 10.1177/13591045221115287

**Published:** 2022-07-19

**Authors:** Emily Hards, Faith Orchard, Sundus Khalid, Clea D’souza, Flora Cohen, Evangeline Gowie, Maria Loades

**Affiliations:** 1 Department of Psychology, 1555 University of Bath, UK; 2 School of Psychology, 1948 University of Sussex, UK; 3 Centre for Academic Child Health, 1980 University of Bristol, UK

**Keywords:** Chronic illness, adolescents, depression, self-evaluation, self

## Abstract

**Objective:**

To conduct a systematic review to establish what is known about the relationship between depression and self-evaluation in adolescents with a chronic illness.

**Methods:**

A systematic search was conducted using MEDLINE, EMBASE, PsycINFO, Web of Science, The Cochrane Library, and hand-searching. We sought to identify primary research that examined both the cross-sectional and longitudinal associations between depression and self-evaluation in adolescents with chronic illness. The search resulted in 8941 retrieved articles that were screened against an inclusion criteria. A total of 4 papers were included in the review. The MMAT used to assess study methodological quality.

**Results:**

A narrative synthesis was conducted, and a summary figure was included. These 4 studies included 236 adolescents aged 9–18 years with depression and either Type 1 Diabetes (T1D), chronic pain, headaches, or Inflammatory Bowel Disease (IBD). The limited existing evidence indicated that that depression was associated with negative self-evaluation in adolescents in some but not all chronic illnesses investigated to date. We also found some evidence that psychological intervention can help to improve self-evaluation, specifically in adolescents with T1D.

**Conclusions:**

More robust studies of the association between self-evaluation and depression in adolescents with a chronic illness is needed, with attention to the nuances of differences between chronic illnesses. The existing evidence indicates that there may be a stronger association in some chronic illnesses. Pilot data suggest that specific psychological therapies may improve self-evaluation, although much more extensive evaluation is needed.

## Introduction

Adolescents are vulnerable to developing depression, with estimates of the cumulative prevalence of Major Depressive Disorder (MDD) ranging from 8% to 20%, for those under the age of 18 (Naicker et al., 2013). In adolescence and young adulthood (ages 15–29), depression is the second leading cause of death via suicide, impacting the quality and length of life (Ferrari et al., 2013; Portzky et al., 2005). Depression in adolescence is also associated with self-harm (Claes et al., 2014; Hawton et al., 2002; Madge et al., 2011) and the onset of other mental health problems in adulthood (Fergusson et al., 2005).

Adolescents with a chronic illness are more vulnerable to mental health problems than those without a chronic illness and are nearly twice as likely to develop a mental health problem including depression (Brady et al., 2020). Given that many terms are used interchangeably in the literature, it is necessary to define what we mean by ‘chronic illness.’ In this paper chronic illness is defined as a “long-term non-communicable medical illness (e.g., diabetes, cystic fibrosis)” that lasts 3 months or more but “still allows for participation in activities of daily living through proper self-management” (p. 334, Zheng et al., 2020; Bernell & Howard, 2016; Compas et al., 2012). Between 15 and 20% of adolescents live with a chronic illness (Jin et al., 2017). Adolescents with a chronic illness are more likely to develop body image issues (Yeo & Sawyer, 2005), have behavioural problems (Newacheck et al., 1991) and are more likely to attempt suicide (Ferro & Boyle, 2013) than adolescents without a chronic illness. Unpredictable exacerbations in symptoms, daily care routines imposed by an illness, treatment, and/or repeated hospitalisation can also exacerbate depression and impact on social activities and relationships with others that are essential for positive development (Zheng et al., 2020).

Negative self-evaluation is one of 10 symptoms listed in in the diagnostic criteria for depression (DSM 5, APA, 2013) and is among the most described symptoms of depression in clinical samples of adolescents (Orchard et al., 2017). A negative view of the self is also a key component of depression within the ‘cognitive triad’ (Beck, 1967). Self-evaluation is defined as how positively or negatively an individual views themselves overall (Beck, 1967; Beck & Alford, 2009) and specifically relates to both positive and negative perceptions of oneself that include traits, characteristics, strengths, and weaknesses (Epstein, 1973; Hattie, 1992). Severity of negative self-evaluation has been found to be associated with severity of depression symptoms in community samples of adolescents (Hards et al., 2020; Orchard et al., 2018) and has been found to predict depression status across clinical and community samples of adolescents (Orchard & Reynolds, 2018). There is also some evidence that self-evaluation can affect depression outcomes over time. For example, research has found that self-evaluation predicted recovery from depression at 3 months and 1 year (Fine et al., 1993).

Self-evaluation is likely to be disrupted in adolescents with a chronic illness due to the burden and management of a chronic illness (Bahrami et al., 2017; Buković et al., 2008; Horky et al., 2017). Children and adolescents with a chronic illness tend to have ‘compromised’ self-evaluation (Ferro & Boyle, 2013) specifically in relation to body image and/or physical appearance compared to healthy controls (Pinquart, 2013a). For example, adolescents with asthma typically report lower scores on domains such as physical appearance and athletic competence compared to healthy peers (Borrego et al., 2005). Adolescents also typically report negative body image and have low appearance evaluation, particularly if management of the illness distorts the physical body such as with stomas, scars and weight gain (Yeo & Sawyer, 2005). Adolescents with a chronic illness also tend to have low self-esteem (Chew et al., 2019), significantly lower than healthy controls (Pinquart, 2013b). However, the relationship between self-esteem and chronic illness is not consistent across chronic illness type or severity. For example a meta-analysis reported that adolescents with chronic fatigue syndrome and chronic headaches had the lowest self-esteem compared to other chronic illnesses (Pinquart, 2013b). However, adolescents with asthma had comparable self-esteem and adolescents with diabetes had higher self-esteem than healthy controls. Therefore, this suggests that the presence of a chronic illness is not always associated with negative self-evaluation.

It is well established that adolescents with a chronic illness are more at risk of depression and in some cases may have a disrupted self-evaluation compared to those without a chronic illness (Ferro & Boyle, 2013; Wrona et al., 2021). However, little is known about how these difficulties co-occur in this population specifically. Furthermore, given the potential for self-evaluation to alter outcomes for depression, it is important to understand how self-evaluation presents in adolescents with depression and chronic illness, and whether self-evaluation is related to recovery from depression. Understanding more about self-evaluation in adolescents with a chronic illness and depression is imperative as this may help to identify support needed for this particularly vulnerable group. This is of particular importance given that research has consistently shown that there is a lack of developmentally appropriate interventions for adolescents with a chronic illness and depression. Despite promising preliminary research, there is a lack of robust specific psychological therapies for this population although some, including Cognitive Behavioural Therapy; CBT (Hickman et al., 2015), e-health interventions (programmes delivered via digital means; Thabrew et al., 2018) and Acceptance and Commitment Therapy; ACT (Kanstrup et al., 2016) have shown promise. Therefore, we conducted a systematic review with the aim of understanding more about self-evaluation in depressed adolescents with a chronic illness. Our review questions were:

(1) Is self-evaluation associated with depression in adolescents with a chronic illness condition both cross-sectionally and prospectively?
(2) Is self-evaluation an important factor in recovery from depression in adolescents with a chronic illness?

## Method

### Literature searches and inclusion criteria

We conducted our review in line with PRISMA guidelines for systematic reviews (see 27-item checklist in the supplementary material (Page et al., 2021). This review was pre-registered with the Open Science Framework (OSF) website link: https://osf.io/e9dgx/. We searched five electronic databases, Web of Science, EMBASE, PsychINFO, Medline, The Cochrane Library in March 2021. Our search was designed to identify any studies that included depression, self-evaluation, adolescents, and chronic illness (see Supplementary Material, Appendix 1 for a full list of search terms). The search strategy and string were primarily based on the searches conducted by (Orchard et al., 2021) in their recent review of self-evaluation in adolescent depression. One author (SK) also conducted hand searches of the first 50 references of the Cochrane library and manually searched the reference lists of any related published literature reviews.

To be included, studies had to be: (1) peer-reviewed publications (1946 (database inception)– 10^th^ March 2021), (2) reporting on primary research and (3) published in English (4) Qualitative or quantitative studies that discussed self-evaluation as a feature of depression or treatment of depression. (5) Participant samples were required to be between 11-24 years old consistent with the World Health Organization’s definition of adolescents and ‘young people’ (World Health Organisation, 2020). (6) Studies had to recruit participants with either a primary diagnosis of depression based on internationally recognised criteria (e.g., DSM, ICD; APA, 2013) or participants with heightened depression symptoms (i.e., scoring above clinical threshold on a validated screening tool). (7) Studies also had to include participants with a chronic illness, chronic disease or physical illness as defined as a “long-term non-communicable medical illness (e.g., diabetes, cystic fibrosis)” that is 3 months or longer, but still allows for participation in activities of daily living through proper self-management” (Bernell & Howard, 2016; Zheng et al., 2020). Only studies that examined a chronic illness meeting the definition mentioned above were included. Studies were excluded if they used adolescents suffering from physical, developmental, and neurological disabilities (e.g., autism).

### Selection process and data collection

Papers were screened for duplicates (FC & EG). Title and abstracts were screened independently using inclusion/exclusion criteria. The first 20% were double screened by EH, SK FC, CS and EG and the remainder was screened by one of the authors. Full text screening was conducted independently by EH, FC, CS and EG, each paper was screened by each author. Any discrepancies during both title and abstract and full text screening were resolved through discussion with a third reviewer (FO). We were unable to access 33 papers included in the full text screening phase despite contacting the corresponding author by email and on ResearchGate; therefore, these papers were excluded. Data were then extracted independently by EH, FC, EG into a database which reported lead author, year of the study, study setting and design, demographic characteristics of the sample and recruitment method, measure of depression, measure and description of self-evaluation, key findings, main limitations, and any information of the treatment/intervention. These data are available on request of the corresponding author. Any discrepancies were discussed and resolved with the help of an additional author (SK).

Assessment of risk of bias in included studies: Quality assessment procedure

The ‘Mixed Methods Appraisal Tool’ (MMAT) (Hong et al., 2018) was used to assess the methodological quality of each included study. This tool includes a different set of criteria for the assessment of five types of study designs: qualitative, quantitative randomised controlled trials, quantitative non-randomised, quantitative descriptive, and mixed methods. It is therefore possible to critically appraise and therefore compare different research designs using one standardised measure (Crowe & Sheppard, 2011). Additionally, the MMAT has good reliability and efficiency and has been used with a range of systematic review topics (Hong et al., 2018).

The methodological quality criteria as defined by the MMAT depending on the research design, was evaluated and the outcome of whether the criteria had been met was indicated by the response of either ‘Yes’, ‘No’ or ‘Can’t tell’. Each paper was independently assessed by two reviewers (EH & FO). Any discrepancies were resolved by discussions between coders and agreement was always reached.

### Data synthesis

We did not conduct a meta-analysis given the broad scope of eligible research designs in the review, i.e., quantitative, and qualitative, and cross-sectional and intervention designs. This would have made these data too heterogenous to be able to pool results together. Instead, a narrative synthesis which summarised the findings of included studies was conducted. The narrative synthesis process was conducted in accordance with guidelines described by Popay et al., (2006). The results of included studies were first synthesised and presented in a table (Table 1). The findings were then discussed and focused around our two research questions; 1. Associations between self-evaluation and depression and 2. Self-evaluation and recovery from depression.

**Table 1.** Summary of included studies.

Authors (year), country	Sample				Healthy comparis-on group	Self terminology	Self measure	Depression measure	Findings	
	Chronic illness (Total N)	Demographic and recruitment	Age range at baseline (years)	Mean age (S.D)					Association between and group differences (statistics)	Longitudinal results
Cross-sectional studies										
Eccleston et al., (2008) United Kingdom	Chronic Pain (*N* = 110). CRPS type 1 (*n* = 51). RAP (*n* = 6), Low back pain (*n* = 7), Chronic headache (*n* = 9), Multiple site idiopathic pain (*n* = 47)	Outpatient Pain Management Clinic (recruited at time of assessment)	11–18	15.1 (1.9)	None	Self-perception and Identity	Self-perception development subscale of BAPQ	CDI	90% adolescents developmentally behind on 1 item of self-perception sub-scale of BAPQ. 51% adolescents developmentally behind in 4 or more items. Perceiving oneself to be less developed in identity - greater pain intensity (β = −0.29, R2 change = 0.104, F = 5.09, *p* < .001). Perceiving oneself to be less developed in identity - greater depression (β = −.026, R2 change = 0.05, F = 5.65, *p* < .05. Strong peer relationships - positive judgment of identity (β = 0.46, R2 change = 0.19, F = 22.98, *p* < .001.	N/A
Guerrero-Ramírez and Cumba-Avilés (2018), Puerto Rico	T1D (*N*=51)	Community sample (Outpatient Diabetes clinic, media, service providers, referrals)	12–17	14.78 (*n*.s)	None	Self-reproach	IVARAC	CDRS-R	Greater self-reproach - higher score on suicidal ideation (r = .36, *p* < .01). Depression highest predictor - suicidal ideation (B = .48, *p* < .001, Higher self-reproach - lower quality of life score (r = .61, *p* < .01)	N/A
Engström (1992), Sweden	IBD (*n* = 24), Tension Headache (*n* = 20), T1D (*n* = 20)	IBD participants - Outpatients in Department of Peadiatrics and Division of Gastroenterology (mothers invited to participate). Tension headache - Primary and secondary school students (questionnaire and random sample of students). T1D - Outpatients in Department of Paediatrics (*n*.s)	9–18	IBD = 16.5 (n.s), Headache = 16.6 (n.s), T1D = 16.4 (*n*.s)	Matched to IBD group for age, gender and choide of subjects at school (*n* = 20)	self-esteem, body image	JTJA	CDI	IBD + Headache groups sig lower total self-esteem than healthy group (F = 8.46, *p* < .05).). Diabetes total self-esteem not sig lower than healthy group (p = n.s). Mental characteristics - Headache and IBD groups sig lower than healthy group (F = 13.52, *p* <.01). Relations with others - IBD and Headache groups sig lower than healthy group (F = 11.85, *p* < .01). Physical characteristics (F = 1.76, *p*>.05), Skills and talents (F = 6.76, *p* > .05), Relationships with parents (F = 3.37, *p* > .05) - no sig difference between groups.	N/A
Intervention study										
Rosselló and Jiménez-Chafey (2006), Puerto Rico	T1D (*N* = 11)	Outpatient Diabetes clinics and members of Diabetes Associations (phone call)	12–16	14.1 (1.3)	None	Self-concept	PHCSCS	CDI		Over 12 weeks, depression (t(10) = 3.00, *p*< .05, self-concept t(10) = −3.72, *p* < .01, self-efficacy for diabetes t(10) = −2.82, *p* < .05 improved

*Notes*. n.s = not specified.

Abbreviations: CRPS, Complex Regional Pain Syndrome Type 1; RAP, Recurrent Abdominal Pain; BAPQ, The Bath Adolescent Pain Questionnaire; CDI, Children’s Depression Inventory; T1D, Type 1 Diabetes Mellitus; PHCSCS, Piers-Harris Children’s Self Concept Scale; IVARAC, Undervaluing/Self-Reproach and Cognitive Alterations Scale; CDRS-R, Children’s Depression Rating Scale-Revised; IBD = Inflammatory Bowel Disease; JTJA, The Jag Tycker Jag Ar (“I think I am”).

## Results

### Study selection

**Figure 1.**
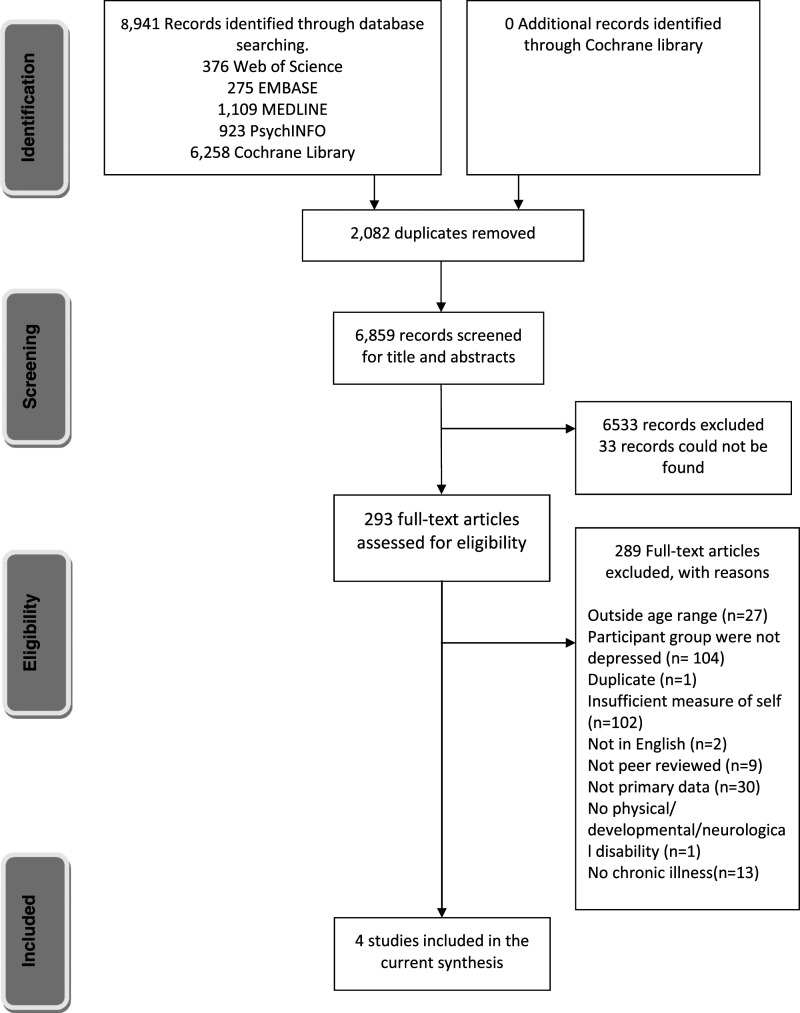
PRISMA flow diagram of included and excluded evidence.

A total of 8941 papers were identified from our initial database searches, hand searches and reference list checking (see Figure 1). After 2082 duplicated were removed 6859 titles and abstracts were screened against the exclusion and inclusion criteria. A further 6566 papers were excluded. A total of 293 papers were full text screened and 289 were excluded because they did not meet the inclusion criteria, for example studies reported adolescents with a chronic illness who were not depressed. In total, 4 papers met our inclusion criteria.

### Study characteristics

All 4 studies were quantitative, three papers were cross-sectional, and one paper was longitudinal (see Table 1). The year of publication ranged from 1992 to 2018. Studies were conducted in the UK (*n* = 1), Puerto Rico (*n* = 2) and Sweden (*n* = 1).

All studies recruited adolescents with similar chronic illnesses, however some studies included adolescents with more than one type of chronic illness. Three studies recruited adolescents with Type 1 Diabetes Mellitus (T1M; Guerrero-Ramírez & Cumba-Avilés, 2018; Engström, 1992; Rosselló & Jiménez-Chafey, 2006). Two studies used adolescents with headaches (i.e., either tension or chronic headache; Engström, 1992; Eccleston et al., 2008). One study recruited adolescents with chronic pain (e.g. Eccleston et al., 2008) and another study (e.g Engström, 1992) used adolescents diagnosed with Inflammatory Bowel Disease (IBD). The mean age of participants ranged from 14.1-16.6 years. The main recruitment method for all participants from all studies was via outpatient clinics.

All four studies included adolescents with heightened depression symptoms. The Children’s Depression Inventory (CDI) was the most used measure to assess depression symptoms. Each study used different self-terminology and different measures of the self.

### Reporting biases: Quality assessment

Each study was given an overall quality assessment score of either 100%, 80%, 60%, 40% or 20% quality criteria met as per guidance from the MMAT. These scores were calculated by dividing the number of criteria met (i.e., a score of ‘yes’) by 4. In this review one paper was rated as high quality (80%), two papers were medium quality (60%) and one paper was low quality (40%). Ratings are displayed in Table 2.

**Table 2.** Quality assessment.

References	3. Non-randomised studies					4. Quantitative descriptive studies					Overall quality score
	3.1	3.2	3.3	3.4	3.5	4.1	4.2	4.3	4.4	4.5	
Eccleston et al., (2008).						Yes	Yes	Yes	Can’t tell	Yes	80%
Rosselló and Jiménez-Chafey (2006)	Yes	Yes	Can’t tell	Can’t tell	Yes						60%
Guerrero-Ramírez and Cumba-Avilés (2018)						Can’t tell	No	Yes	Can’t tell	Yes	40%
Engström (1992)						Yes	Yes	Can’t tell	Yes	No	60%

### Summary of studies

#### Associations between self-evaluation and depression

Three studies investigated depression and self-evaluation in adolescents with a chronic illness (e.g.Engström, 1992; Rosselló & Jiménez-Chafey, 2006; Eccleston et al., 2008). Only one study (Eccleston et al., 2008) reported associations between self-evaluation and depression and was rated as high quality (80%). Another study reported associations between self-evaluation and suicidal ideation (Guerrero-Ramírez & Cumba-Avilés, 2018) and was low quality (40%). Specifically, this paper met our inclusion criteria however it didn’t directly address the question of interest (i.e., examine associations between depression and self-evaluation). Another paper (e.g. Engström, 1992) described the relationship between self-evaluation and chronic illness and depression and chronic illness separately and was medium quality (60%).

Two studies reported a corrrelation between self-evaluation and depression/suicidal ideation in adolescents with a chronic illnesses (Eccleston et al., 2008; Guerrero-Ramírez & Cumba-Avilés, 2018). Specifically, one study (Guerrero-Ramírez & Cumba-Avilés, 2018) examined adolescents with T1D and reported that greater self-reproach (i.e., more negative self-evaluation) was associated with increased frequency of suicidal ideation in adolescents with T1D. (r = .36). Additionally, more negative self-evaluation was associated with lower quality of life (r = .61). In another paper (i.e., Eccleston et al., 2008) with adolescents experiencing chronic pain, factor analysis identified three factors of self-perception of development: independence, emotional adjustment, and identity formation. All factors were negatively associated with depression (r = −.31, −.32 & −.35 respectively) indicating that judging oneself as less independent, less emotionally adjusted and less developed in identity was associated with elevated depression symptoms. Judging oneself as developmentally behind peers across all factors of self-perception was also associated with increased pain intensity.

Two of the three studies (e.g., Engström, 1992; Eccleston et al., 2009) also examined specific components of self-evaluation in adolescents with a chronic illness and described mixed results. Engström (1992) reported that adolescents with IBD and headaches had significantly lower total self-esteem than healthy controls (*p* < .05). Specifically, adolescents with IBD and headaches had significantly lower scores in self-evaluation domains such as mental characteristics (*p* < .01) and relationships with others (*p* <.01) compared to healthy adolescents. However, there were no significant differences across other domains such as physical appearance and relationships with parents. Also in this study, authors reported that adolescents with T1D had comparable total self-esteem scores to healthy adolescents without a chronic illness and there were no significant differences in any self-evaluation domains. Similarly, in another paper (Eccleston et al., 2008) adolescents with chronic pain were asked to estimate feelings of being behind or ahead in relation to domains of self-evaluation. Adolescents said they felt behind in 5/11 domains including progress at school, confidence around people, doing things without parents around, independence and going on dates. However, adolescents judged themselves as comparable to their peers across 5/11 self-evaluation domains including future plans, choosing own clothing and personal items, development of identity and handling their feelings. Adolescents also judged themselves as ahead of their peers in relation to dealing with problems.

One cross-sectional study (e.g., Eccleston et al., 2008) also reported a potential protective mechanism in relation to self-evaluation in adolescents with a chronic illness. It was found that strong peer relationships were positively correlated with higher self-evaluation across three domains of self-evaluation including judgement of independence (r = .39), emotional adjustment (r = .36) and identity development (r = .47).

### Self-evaluation and recovery from depression

Only one study examined the prospective relationship between self-evaluation and depression in adolescents with a chronic illness (Rosselló & Jiménez-Chafey, 2006). This paper was rated as medium quality (60%)*.* In this study, 11 adolescents with both T1D and symptoms of depression received group CBT over a period of 12 weeks. Self-evaluation and depression symptoms among other variables were assessed both pre and post treatment. Self-evaluation (t = −3.72, *p* < .01) and depression (t = 3.00, *p*<.05) scores improved post-treatment.

## Discussion

This systematic review found that few studies have investigated the relationship between self-evaluation and depression in adolescents with a chronic illness, suggesting that this is an under-researched area. Despite this, there is some tentative evidence that negative self-evaluation is associated with depression in adolescents with some types of chronic illness. These findings are important given that research suggests that for those with a chronic illness, self-evaluation is likely to be more negative due to the management and burden of the illness (Bahrami et al., 2017; Buković et al., 2008; Horky et al., 2017). The negative correlation between self-evaluation and depression is also consistent with research with adolescents without a chronic illness (Hards et al., 2020; Orchard et al., 2018).

Findings from this systematic review also suggest however that disruptions to self-evaluation are not consistent across *all* chronic conditions in adolescents. Different chronic conditions were associated with a more severe impact on self-evaluation than others. For example, adolescents with IBD and headaches had lower total self-esteem and lower scores across self-evaluation domains such as mental characteristics than healthy controls. Whereas, adolescents with T1D had both comparable total self-esteem scores and self-evaluation scores across all domains measured (Engström, 1992). These results are important as they highlight that different chronic illnesses may impact self-evaluation in different ways. Specifically, previous research has highlighted that those with illnesses which are more visible such as cerebral palsy have lower self-esteem than adolescents with less visible illness such as T1D. This is because a visible illness is likely to have an impact on an adolescent’s evaluation of their physical appearance (Pinquart, 2013b). However, it may be the case that the management of a chronic illness rather than the type of chronic illness itself is an important factor. For example, greater perceived symptom severity is associated with lower self-esteem (Juth et al., 2008) and other factors such as a lack of control or predictability of the illness are all likely to impact on self-esteem (Bedrov & Bulaj, 2018). Additionally, these factors are also likely to impact on opportunities to participate in activities with peers, during a time when peer relationships are of heightened importance to adolescents (Goossens, 2018). For example, those with a chronic physical illness are somewhat lonelier than their peers without a chronic illness (Maes et al., 2017). This is also likely to have negative implications on self-evaluation (Geukens et al., 2020). Future research would therefore benefit from examining the relationship between self-evaluation and depression in *specific* chronic illnesses, while accounting for perceived self-management and controllability of chronic illnesses. Taking this more individualised approach would improve our understanding of depression and have implications for the assessment and treatment of depression in this vulnerable population.

In relation to the second research question of this study i.e., self-evaluation and recovery from depression, there was some evidence that psychological intervention might help to improve self-evaluation and depression symptoms in adolescents with a chronic illness. Specifically, a group CBT treatment designed to reduce depression symptoms and improve glycaemic control showed promising pilot data in adolescents with T1D and led to improvements in self-evaluation (Rosselló & Jiménez-Chafey, 2006). However, this was the only study we found that reported a psychological intervention and longitudinal results. This lack of robust evidence is problematic and highlights an urgent need for much more extensive investigation. There was also some evidence that strong peer relationships were associated with more positive self-evaluation in adolescents with chronic pain (Eccleston et al., 2008). Therefore, peer relationships may be an important protective factor of self-evaluation in this population. This is consistent with evidence which suggests that social support is an important factor in the management of a chronic illness. For example, in a sample of adults with Cystic Fibrosis (CF) social support was associated with fewer mental and physical health symptoms, reduced treatment burden and a better overall perception of their health over time (Flewelling et al., 2019). However, more research is needed using adolescent sample that also considers the impact of depression.

The results of this review may have important implications for the assessment and treatment of depression in adolescents with a chronic illness. Negative self-evaluation is a commonly identified symptom of depression in adolescents and is typically assessed via a structured diagnostic interview and/or questionnaires which evaluate global positive and negative self-evaluation. However, the findings of this review suggest that some adolescents with both depression and a chronic illness may not describe a negative self-evaluation in relation to *all* domains of their self. Instead, it may be the case that they retain a positive self-evaluation in relation to specific domains. For example, adolescents with IBD and headaches were found to retain positive self-evaluation in relation to their physical appearance and family relationships (Engström, 1992). Therefore, despite pronounced negative self-evaluation, there may be components that remain positive. These protected positive domains of self-evaluation could be an important focus of therapy as they may be easier to endorse while depressed and could offer a basis for improving self-evaluation in adolescents with a chronic illness. However, this review also found evidence to suggest that not all adolescents with a chronic illness describe negative self-evaluation. Therefore, this may not be a common symptom of depression in some chronic illness populations such as adolescents with T1D and therefore therapy that is focused on improving self-evaluation may not be appropriate. Future research should urgently examine self-evaluation in specific chronic illnesses to ascertain whether this is the case.

### Strengths and limitations

This was the first systematic review to examine the relationship between self-evaluation and depression in adolescents with a chronic illness. Screening was robust, hand-search strategies were used and authors were contacted for additional information and access to their papers. However grey literature was not searched as we only included peer reviewed papers as a proxy sign of rigour, and only publications published in English were included, therefore it is possible that some studies may have been missed.

In this review, we focused exclusively on the co-occurrence of both self-evaluation and depression in adolescents with a chronic illness. In doing so, we excluded evidence relating to the wider adolescent population (i.e., those without a chronic illness). Whilst there are likely to be considerable overlaps with the latter, the experience of chronic illness which distinguishes this group may particularly impact on self-evaluation e.g., Bahrami et al., (2017); Buković et al., (2008); Horky et al., (2017). Furthermore, as this group are often seen clinically in paediatric health settings, it was important to consider this group separately. Thus, our review adds to and should be considered in conjunction with existing literature inclusive of all adolescents.

The overall quality of included studies was fair; however only one paper was identified as high quality. There were also common limitations of all included studies. Not all papers included a comparison group and therefore it is difficult to ascertain the cross-sectional relationship between self-evaluation and depression in adolescents with a chronic illness without a baseline. Additionally, the only longitudinal study reported in this paper did not include a control group and therefore it is difficult to reliably draw conclusions about the prospective relationship between self-evaluation and depression in adolescents with a chronic illness and the impact of this intervention. Also, only four studies met the inclusion criteria and were reviewed in this manuscript. This lack of range is problematic and makes it difficult to draw reliable conclusion given that only a small number of different types of chronic illnesses (e.g., T1D, chronic pain and headaches) were reported in the included papers. This reflects a small sample size in respect to both the amount of disease categories and the number of participants included in the studies. Given this, we were not able to differentiate between different types of chronic illness. Therefore, the chronic illness types represented in this review are diverse and it is possible that the illness experience, the psychological processes, and the physiological processes may be vastly different. It will be important for future reviews to take a more nuanced approach to differentiate between adolescents’ different types of chronic illness which cluster together more closely in terms of pathology and presentation, for example, those with persistent physical symptoms such as chronic pain and chronic fatigue specifically.

## Conclusion

This systematic review highlights that there is some tentative evidence to suggest that negative self-evaluation is associated with depression in adolescents with a chronic illness. However, there is a lack of robust evidence of the specific relationship between self-evaluation and depression, both cross-sectionally and longitudinally, in adolescents with chronic illnesses and much more research is urgently needed. This review also found evidence that a specific psychological intervention may help to improve self-evaluation in adolescents with T1D, although more robust trials are needed.

## Supplemental Material

Supplemental Material - Self-evaluation and depression in adolescents with a chronic illness: A systematic review Click here for additional data file. Supplemental Material for Self-evaluation and depression in adolescents with a chronic illness: A systematic review by E Hards, F Orchard, S Khalid, C D’souza, F Cohen, E Gowie and ME Loades in Clinical Child Psychology and Psychiatry
